# Understanding the impact of colorectal cancer education: a randomized trial of health fairs

**DOI:** 10.1186/s12889-015-2499-2

**Published:** 2015-11-30

**Authors:** Katherine J. Briant, Lei Wang, Sarah Holte, Adriana Ramos, Nathan Marchello, Beti Thompson

**Affiliations:** Fred Hutchinson Cancer Research Center, Public Health Sciences, 1100 Fairview Avenue North, M3-B232, Seattle, WA 98109 USA; Departments of Biostatistics and Global Health, University of Washington, 4333 Brooklyn Ave NE, Seattle, WA 98105 USA; Fred Hutchinson Cancer Research Center, Center for Community Health Promotion, 320 North 16th Street, Sunnyside, WA 98944 USA

**Keywords:** Colorectal cancer, Colorectal cancer screening, Health education, Health fairs, Educational tools, Inflatable colon, Hispanics, Promotores

## Abstract

**Background:**

Regular screening for colorectal cancer (CRC) reduces morbidity and mortality from this disease. A number of factors play a role in the underutilization of CRC screening; populations with the lowest CRC screening rates are least likely to be aware of the need for screening or have knowledge about screening options. The overall purpose of this project was to assess two methods for increasing knowledge about CRC in a health fair context: one, by using a health educator to provide CRC information at a table, or two, to provide a tour through a giant inflatable, walk-through colon model with physical depictions of healthy tissue, polyps, and CRC.

**Methods:**

We participated in six community health fair events, three were randomized to incorporate the use of the inflatable colon, and three used a standard display table method. We used a pre/post-design to look for changes in knowledge about CRC before and after participating in a health fair. We examined descriptive statistics of participants using frequencies and proportions. McNemar’s test for paired binary data was used to test whether there were significant differences in the distribution of correct answer percentage from pre to post and from pre to follow up. Linear regression (GEE) was used to investigate whether there was a significant difference in the change from pre- to post-intervention in the percentage of correct answers on knowledge of tests available to detect CRC and awareness of risk factors for CRC between participants at sites with the inflatable colon compared to participants at sites without the inflatable colon.

**Results:**

Participants (*n* = 273) were recruited at the six health fairs. Participants in health fairs with the inflatable colon had higher knowledge at post-test than participants in health fairs with tabling activities, that is, without the inflatable colon; however, the difference was not significant. One month follow-up after each health fair showed virtually no recollection of information learned at the health fairs.

**Conclusions:**

The use of an inflatable colon may be an innovative way to help people learn about CRC and CRC screening; however, it is not significantly more effective than conventional table display methods. Further research is needed to associate intention to obtain screening after touring the inflatable colon with actual screening. Future research could explore ways to better retain knowledge at long-term follow-up.

## Background

The United States (US) Preventive Services Task Force, the American Cancer Society and the American College of Physicians have established national guidelines that recommend colorectal cancer (CRC) screening for average-risk adults starting at age 50 [1]. Although there have been general increases in CRC screening in the last two decades [2], there are disparities in CRC screening, as well as in CRC incidence and mortality [3]. Research indicates that CRC screening is especially low in minority and low socio-economic status (SES) groups; some research suggests that these populations have both low awareness of CRC and low knowledge about CRC screening [4–7].

Health fairs are a common health promotion strategy used in community settings to disseminate information about public health topics to a large segment of a population. Health professionals, or trained organizational representatives and volunteers sit at a table and offer educational materials, interactive activities, and are available to answer questions. When resources are available, health screening tests such as oral exams, blood pressure checks, cholesterol tests, diabetes tests, HIV tests, and mammography may also offered at these health fair events [8]. There is inconclusive evidence regarding the effectiveness of health screenings done at health fairs. One study in 1985 found that screenings may generate false positives in healthy people, and false negative results may provide a false sense of security for people at risk for disease [9]. Interestingly, a study published in 1991 found that among 303 adult health fair participants, 47 % reported that obtaining screening tests was the sole reason for attending the event [10]. Another study published in 2006 found that screenings may offer financial value for people with little or no insurance [11]. Nevertheless, most recently, community health fairs that offer screenings have been found to be a culturally appropriate way to reach underserved Hispanics [12].

Studies looking at the impact of health fairs have been few. In 2001, one published study did a formative evaluation of the planning and implementation of a health fair and found the event to be a success. Unfortunately, outcome evaluation was beyond the scope of that project [13]. In 2005, a study found lifestyle changes made among participants in rural farm health fairs [14]. Most recently, in 2015, senior wellness fairs were found to be an effective tool to help students obtain skills and knowledge in providing health promotion information to older adults [15].

The Center for Community Health Promotion (CCHP) of the Fred Hutchinson Cancer Research Center (FHCRC) participates in many health fairs throughout three counties in Eastern Washington. CCHP *promotores* offer instruction about CRC and CRC screening at these events using an inflatable colon. When the inflatable colon cannot be accommodated at an event, the *promotores* use more conventional materials, such as flip charts, tabletop displays, brochures, and videos. The inflatable colon has been demonstrated to be efficacious in increasing knowledge and intention to be screened, as well as actual screening behavior using a one group pre-test/post-test design in different geographic areas of the US [16–18]. Based on findings from a local prior study where we learned that the inflatable colon was an innovative way to learn about CRC [16], the project team was interested in learning about the success of the inflatable colon relative to the more conventional materials in increasing CRC awareness and knowledge as well as intention to be screened. The overall purpose of this project was to assess two different methods for increasing knowledge about CRC in a health fair context: one, by using a health educator to provide CRC information at a table, or two, to provide a tour through a giant, inflatable, walk-through colon model with physical depictions of healthy tissue, polyps, and CRC.

## Methods

### Setting

One of 23 Community Network Program Centers (CNPCs) in the US, the CCHP of the FHCRC is located in a rural, agricultural area in Eastern Washington State east of the Cascade Mountain range. Many communities in this area are majority-minority (Hispanic) towns with those of Hispanic ethnicity (primarily Mexican) forming 67 % of the population; the population in general is underserved in terms of poverty, educational status, and having insurance [19]. The CCHP seeks to reduce health disparities in this geographic area, especially among low socio-economic status Hispanics. Based on findings from two Town Hall Forums conducted by the CCHP in April 2011, community members reported being most concerned about CRC compared to other cancer sites. Thus, the CCHP decided to focus on CRC education and raising awareness about CRC screening in the first years of the CNPC. The six communities in which the health fairs were held are very similar with a high proportion of individuals (60 to 70 %) of Hispanic origin.

### Intervention

After discussion with community members, the CCHP purchased a giant colon; this is a walk through inflatable colon (10 ft high, 12 ft wide, and 20 ft long) that contains simulated normal tissue, polyps, cancers, and advanced cancers. Six display signs inside the colon explain the progression of cancer from normal tissue to advanced stage cancer and highlight the importance of screening and early detection of CRC. Signs were created in English and Spanish. The inflatable colon is used at community events to increase awareness and knowledge of CRC. Tours through the colon are led by trained *promotores* (lay health workers) who are staff of the CCHP. The 12-min tours emphasize what can be done to reduce the risk of CRC.

CCHP staff worked with community partners to participate in the six community health fairs between May and August of 2013. The six health fairs were randomized so that the inflatable colon appeared at three of those health fairs, while the other three held the standard tabletop information and materials. Trained CCHP *promotores* participated in the health fairs by either staffing a table with CRC and CRC screening information in English and Spanish, or facilitating a tour of the inflatable colon. A description of the colon tours is available elsewhere; briefly, *promotores* led tours in the giant colon explaining the advantages of CRC screening [16]. At the display tables, *promotores* talked to participants about CRC addressing topics such as risk factors and the importance of screening tests.

### Measurement

As community members arrived at each health fair, adults 18 and older were invited to participate in this evaluation. If they were interested in participating, individuals were given a pre-numbered packet containing a pre- and post-questionnaire. The packet also included a passport to keep track of the tables and displays they visited at the health fair. Participants in this evaluation completed the pre-questionnaire when they picked up their packet. When they handed in their passport, they completed the post-questionnaire. Participants completed pre- and post- pencil and paper questionnaires in their language of choice (English or Spanish). CCHP *promotores* were available to help read questionnaires to participants who needed assistance. When they returned the post-questionnaire, they were given a water bottle as an incentive. When participants completed the post assessment questionnaire, they were asked if they were interested in completing a one month follow-up questionnaire via the telephone. If they consented, CCHP *promotores* followed up with a phone call approximately one month later to ascertain whether the participant had taken steps to be screened.

The protocol, as well as participant consent language, and all questionnaires for this intervention, were approved by FHCRC Institutional Review Board.

### Study measures

We measured participants’ knowledge of CRC and CRC screening, past screening behavior, and access to health care. Demographic variables collected on the pre-test, included gender, age, race/ethnicity, whether they had health insurance, and whether they had a regular health clinic. Awareness of screening was assessed by pre-test and post-test responses to yes/no questions where respondents were asked if they knew what a fecal occult blood test (FOBT), sigmoidoscopy and colonoscopy were. Knowledge was further assessed with nine yes/no questions at pre-test and post-test; these questions asked the respondents if they thought most patients can survive CRC if it is found early and removed, and the remaining eight questions asked if they knew whether factors were associated with an increased risk of CRC (getting older, a diet that doesn’t have many fruits and vegetables, a family history of CRC, a diet high in fat and low in fiber, smoking, having type 2 diabetes, lack of physical activity, and being overweight or obese).

Study data were collected and managed using Research Electronic Data Capture (REDCap) electronic data capture tools hosted at FHCRC [20]. REDCap is a secure, web-based application designed to support data capture for research studies, providing 1) an intuitive interface for validated data entry; 2) audit trails for tracking data manipulation and export procedures; 3) automated export procedures for seamless data downloads to common statistical packages; and 4) procedures for importing data from external sources.

### Analysis

We first examined descriptive statistics of participants using frequencies and proportions. The percentage of correct answers for the twelve awareness/knowledge questions are shown for pre (entrance) and post (exit) test by randomized location (sites with inflatable colon vs. sites without inflatable colon) was calculated. McNemar’s test for paired binary data was used to test whether there were significant differences in the distribution of a correct answer on individual questions from pre- to post-test and from pre-test to follow up (Table 2).

We used linear regression to investigate whether there was a significant difference in the change in percentage of correct answers from pre- to post-test (Table 3) between participants at sites with the inflatable colon compared to participants at sites with Tables (i.e., without the inflatable colon). In this analysis, the percentage correct for the awareness of screening tests available and the percentage correct for knowledge of risk factors for CRC was calculated for each participant pre-and post-intervention, and the difference in these percentages from pre- to post-intervention was used as the dependent variable in a linear regression model, with covariate for randomization status (inflatable colon vs information tables). Generalized estimating equations (GEE) were used to account for intra-site correlations.

The difference in the change in correct answers for both knowledge and awareness were estimated using the linear formula: DP = β0 + β1*χ1 + ɛ, where DP is the change in the percentage of questions answered correctly from pre- to post-intervention and χ1 is an indicator variable coded “1” for participants who received information via the inflatable colon and “0” for those who received information from tables. Hence, β1, is the coefficient of interest for evaluating the effect of receiving information on CRC via the inflatable colon. All statistical tests were two-tailed with a significant level at 0.05. Analysis was performed with SAS for Microsoft Windows (version 9.3, SAS Institute Inc.).

## Results

During the four month intervention, 273 participants completed pre- (entrance) questionnaires, 247 participants finished pre- and post- (exit) questionnaires, and 205 finished pre-, post- and one month follow-up questionnaires. Characteristics of the 273 participants are presented in Table 1. The 273 participants were recruited from the six health fairs: three health fairs with the inflatable colon (*n* = 134, 49.1 %) and three health fairs without the inflatable colon, that is, only with information tables (*n* = 139, 50.9 %). The majority of participants were female (208, 76.2 %). Of the four age categories, the majority of participants were 40 to 49 years old (84, 30.8 %).

**Table 1:** Participant demographic characteristics

Characteristic	With colon^a^ (134)	With tabling^a^ (139)	Total (273)
	*n* (%)	*n* (%)	*n* (%)
Gender			
Female	97 (72.4)	111 (79.9)	208 (76.2)
Male	37 (27.6)	28 (20.1)	65 (23.8)
Age			
< 30	26 (19.4)	24 (17.3)	50 (18.3)
30-39	40 (29.9)	32 (23)	72 (26.4)
40-49	35 (26.1)	49 (35.3)	84 (30.8)
50+	33 (24.6)	34 (24.5)	67 (24.5)
Regular Health Clinic			
Yes	111 (82.8)	126 (90.7)	237 (86.8)
No	23 (17.2)	13 (9.4)	36 (13.2)
Health Care Plan/Insurance			
Private	17 (12.7)	21 (15.2)	38 (14)
Basic Health Plan	13 (9.7)	9 (6.5)	22 (8.1)
Medicare	6 (4.5)	8 (5.8)	14 (5.1)
Medicaid, Coupons, VA, IHS	7 (5.2)	18 (13)	25 (9.2)
None	91 (67.9)	82 (59.4)	173 (63.6)
Ethnicity			
NHW	3 (2.3)	2 (1.5)	5 (1.9)
Hispanic	129 (97.7)	136 (98.6)	265 (98.1)

^a^Does not include missing responses

In Table 2, familiarity with individual CRC knowledge questions was examined for the 247 participants who completed pre- and post-tests. Among participants at health fairs without the inflatable colon, there was a significant improvement in knowledge of CRC (answering incorrect at pre-test to answering correct at post-test) for three questions: “Know Fecal Occult Blood Test (FOBT) is available for CRC” (*p* < 0.01), “Know Sigmoidoscopy is available for CRC” (*p* < 0.01), and “Know Colonoscopy is available for CRC” (*p* < 0.01). Among participants at health fairs with the inflatable colon, there was a significant improvement in knowledge of CRC for six questions: “Know Fecal Occult Blood Test (FOBT) is available for CRC” (*p* < 0.01), “Know Sigmoidoscopy is available for CRC” (*p* < 0.01), “Know Colonoscopy is available for CRC” (*p* < 0.01), “Getting older increases the risk of CRC” (*p* < 0.01), “A diet that doesn’t have many fruits and vegetables increases the risk of CRC” (*p* = 0.02), and “A family history of colorectal cancer increases the risk of CRC” (*p* = 0.01).

**Table 2:** Pre-test, post-test and 1-month follow up percentage of correct answers

Knowledge questions (% responding correctly)	With tabling			With colon		
	Pre	Post	Follow-up	Pre	Post	Follow-up
Know Fecal Occult Blood Test (FOBT) is available for CRC	19.42	34.88^a^	17.43	21.64	56.78^a^	21.88
Know Sigmoidoscopy is available for CRC	16.55	31.01^a^	13.76	14.93	49.15^a^	12.5
Know Colonoscopy is available for CRC	21.58	38.76^a^	21.1	26.12	54.24^a^	27.08
Most patients can survive CRC if it is found early and removed	95.45	96.88	95.37	96.64	100	97.87
Getting older increases the risk of CRC	69.06	69.77	68.81	69.4	84.75^a^	78.13
A diet lack of fruits and vegetables increases the risk of CRC	79.86	79.84	85.32	78.36	88.98^a^	90.63^b^
A family history of colorectal cancer increases the risk of CRC	76.26	82.17	74.31	82.84	93.22^a^	81.25
A diet that is high in fat and low in fiber increases the risk of CRC	82.73	89.15	85.32	90.3	94.87	88.42
Smoking increases the risk of CRC	78.42	83.72	77.06	82.84	87.29	84.38
Having type 2 diabetes increases the risk of CRC	65.22	72.09	64.22	72.39	80.51	63.54
Lack of physical activity increases the risk of CRC	79.14	84.5	83.49	83.58	87.18	89.58
Being overweight or obese increases the risk of CRC	83.45	87.6	87.16	87.31	90.68	88.54

^a^The distribution of percentage of correct answers at pre and post-test is significantly different at level p < 0.05, based on McNemar’s test for paired binary data

^b^The distribution of percentage of correct answers at pre-test and 1-month follow-up is significantly different at level p < 0.05, based on McNemar's test for paired binary data

The results of the linear regression analysis for the change from pre- to post-intervention in the percentage of those answering awareness and knowledge questions correctly are described in Table 3. For participants who received information using the inflatable colon, the increase in the percentage correct on the awareness of screening tests available was 33 % compared to 15 % for those at tabling. Although the difference was almost double for the inflatable colon group, the difference between the two groups in the change in the awareness gain was not statistically significant (*p* value = 0.17) based on robust standard errors from GEE estimates to account for intra-site correlation.

**Table 3:** Pre- and post-test change (percentage correct) in awareness and knowledge

	Tabling			Colon			p value^a^
	Pre	Post	Change	Pre	Post	Change	
Awareness of CRC Screening	0.19 (n = 139)	0.35 (*n* = 129)	0.15 (*n* = 129)^b^	0.21 (*n* = 134)	0.53 (*n* = 118)	0.33 (*n* = 118)^b^	0.17
Knowledge of CRC risk factors	0.79 (n = 131)	0.83 (*n* = 128)	0.02 (*n* = 122)^b^	0.84 (n = 119)	0.90 (*n* = 115)	0.07 (*n* = 104)^b^	0.09

^a^For difference in change in percentage correct from pre- to post-test by tabling vs. colon

^b^Percentage change not equal to post-pre due to different number of participants at pre- and post-test visits (percentage correct)

In the analysis of change in knowledge of risk factors for CRC, participants who received information using the inflatable colon showed an increase in the percentage correct of 7 % compared to participants who received information through tabling, where the increase in the percentage correct on knowledge of risk factors for CRC questions was 2 %. Again, however, the difference between the two groups in the change in knowledge of 5 % was not statistically significant (*p* value = 0.09) based on robust standard errors from GEE estimates.

## Discussion

In this randomized study, we demonstrated that the use of an inflatable colon increases knowledge about CRC and CRC screening; and is more effective at increasing knowledge than conventional materials, such as flip charts, tabletop displays, brochures, and videos. However, these observed differences were not statistically significant, possibly due to sample size. We also learned that awareness of CRC screening methods increased by a difference of 18 %, but this also was not statistically significant. While there are trends towards the benefit of the inflatable colon our sample size may not have been sufficient to detect significant differences. Intra-site correlation may have reduced our power to detect significant differences between the two groups. Future studies with more participants per study site (e.g. health fair) or more sites would improve power to detect differences in the use of the inflatable colon to educate individuals about CRC.

This study adds to the body of knowledge that educational interventions which vary in the type of learning methods (visual, text and audio) are no more effective at increasing comprehension and recall than interventions that only include written/text materials [21]. In this small study of two clusters, both methods had similar outcomes in terms of awareness of CRC screening methods and in CRC knowledge overall [22–24]. We hypothesized that the inflatable colon, a visual and interactive display, would be more likely to increase CRC knowledge and awareness than an information table with conventional materials. However, even when combined with a walk-through tour, the inflatable colon was not significantly more effective compared to conventional materials.

Data from the one month follow-up questionnaires indicated that it was irrelevant how participants learned about CRC, neither arm (inflatable colon or tabletop information and materials) seemed to retain the knowledge one month after the health fair event. When examining the significance in knowledge after one month from pre- to follow-up, none of the questions had significant improvement except one, “A diet that doesn’t have many fruits and vegetables increases the risk of CRC” (*p* < 0.01). This was somewhat discouraging. One reason that may contribute to this is the use of a brief telephone call for the follow-up questionnaire, compared to *promotor(a)*-led personal questionnaires at the pre- and post-test stages. Another reason may be that the one-time, short interaction with *promotores*, either through the inflatable colon tour, or at an information table, is not long enough to enable participants to retain long-term understanding about CRC. It may be that more exposure to CRC information increases knowledge retention and likelihood to be screened [25]. It may also be that an intervention after participation in the health fair may be needed to have an impact on knowledge and behavior [26, 27]. Another explanation may be that not only health literacy, but also participants’ cognitive abilities, such as working memory and long-term memory, affect their ability to recall information [28]. Given that this population is of low socioeconomic status, it may be that there cognitive abilities are affected by the stressors in their daily lives.

In the US, racial differences in practices, knowledge, and barriers related to CRC screening exist [29]. There is evidence to show that literacy and knowledge regarding cancer may affect participation in prevention [30–32]. When compared to non-Hispanic whites, minorities are more likely to have inaccurate knowledge and beliefs regarding CRC, as well as increased perceived barriers regarding CRC screening [33]. More research is needed regarding interventions that address barriers to CRC screening among Hispanics, the fastest growing population in the US. It is imperative to reinforce the importance of CRC screening and encourage age-eligible participants to obtain CRC screening, but equally important to address barriers to screening [34, 35]. Barriers to colorectal cancer among Hispanics include factors such as fatalism about cancer survival, fear of the test, cost, low literacy and low level education, lack of awareness about screening, and lack of provider recommendation [36, 37]. With the advent of healthcare reform in the US, cost may become less of an issue for documented Hispanics.

### Strengths and Limitations

A strength of this study is the randomized control design. The study also has some limitations. There may have been bias in the self-selection of the sample; those choosing to attend a health fair may differ from the general population. We had participants younger than 50 years of age. We asked participants if the health fair helped them decide to visit the doctor for a check-up or health screening, but we do not know if intention predicted actual behavior. Although we asked about CRC screening history among study participants, data was self-reported. We learned in a previous study that if education is coupled with access to a free fecal occult blood test, participants are very likely to comply with CRC screening [16]; however for this study, we did not have resources to offer free or low-cost CRC screening for participants 50 and older who had not been screened. Finally, it is worth mentioning that the time from pre- to post-questionnaire at the health fairs ranged between 30–120 min. Information may have still been “fresh” in participants’ minds which would make it easier to recall information at the post-questionnaire compared to the one-month follow-up questionnaire.

## Conclusions

The use of an inflatable colon or tabling to instruct participants at health fairs are both effective in changing participants awareness of CRC screening and in knowledge about CRC. There were more significant changes in CRC knowledge among participants who participated in an inflatable colon tour, compared to participants who did not; however, overall, the differences were not significant. Further research is needed to associate intention to obtain CRC screening after learning about CRC with actual CRC screening. Future research could explore ways to better retain CRC knowledge at long-term follow-up.
